# Explainable DCNN Decision Framework for Breast Lesion Classification from Ultrasound Images Based on Cancer Characteristics

**DOI:** 10.3390/bioengineering11050453

**Published:** 2024-05-02

**Authors:** Alaa AlZoubi, Ali Eskandari, Harry Yu, Hongbo Du

**Affiliations:** 1 School of Computing, University of Derby, Derby DE3 16B, UK; a.eskandari@derby.ac.uk (A.E.); h.yu@derby.ac.uk (H.Y.); 2 School of Computing, The University of Buckingham, Buckingham MK18 1EG, UK; hongbo.du@buckingham.ac.uk

**Keywords:** deep convolutional neural networks, visual explanations, saliency maps, cancer recognition, breast cancer, ultrasonography, cancer characteristics

## Abstract

In recent years, deep convolutional neural networks (DCNNs) have shown promising performance in medical image analysis, including breast lesion classification in 2D ultrasound (US) images. Despite the outstanding performance of DCNN solutions, explaining their decisions remains an open investigation. Yet, the explainability of DCNN models has become essential for healthcare systems to accept and trust the models. This paper presents a novel framework for explaining DCNN classification decisions of lesions in ultrasound images using the saliency maps linking the DCNN decisions to known cancer characteristics in the medical domain. The proposed framework consists of three main phases. First, DCNN models for classification in ultrasound images are built. Next, selected methods for visualization are applied to obtain saliency maps on the input images of the DCNN models. In the final phase, the visualization outputs and domain-known cancer characteristics are mapped. The paper then demonstrates the use of the framework for breast lesion classification from ultrasound images. We first follow the transfer learning approach and build two DCNN models. We then analyze the visualization outputs of the trained DCNN models using the EGrad-CAM and Ablation-CAM methods. We map the DCNN model decisions of benign and malignant lesions through the visualization outputs to the characteristics such as echogenicity, calcification, shape, and margin. A retrospective dataset of 1298 US images collected from different hospitals is used to evaluate the effectiveness of the framework. The test results show that these characteristics contribute differently to the benign and malignant lesions’ decisions. Our study provides the foundation for other researchers to explain the DCNN classification decisions of other cancer types.

## 1. Introduction

Breast cancer is one of the most common cancers affecting women globally [1]. Magnetic resonance imaging (MRI), computerized tomography (CT), and ultrasonography (US) have become crucial imaging modalities that are frequently used today to screen and help in diagnosing breast lesions. Compared to MRI and CT, US is nonradiative, inexpensive to maintain, and widely available [2]. However, US images are adversely affected by speckle noise and artefacts, making lesion classification challenging. Because US scans are performed by radiologists with varying levels of experience, observing signs of malignancy in lesions during the scan is far from objective [3]. The American College of Radiology’s Breast Imaging-Reporting and Data System (ACR BI-RADS) is a standardized description system that is widely followed by radiologists to identify and classify breast lesion characteristics from US images [4].

Deep learning techniques have been widely used to assist medical diagnosis. In particular, CNNs have achieved outstanding performance in breast lesion classification [5], detection [6], and segmentation [7] by capturing and learning discriminative features such as color, texture, and shape from images. Several deep learning approaches have been designed, customized, and developed to classify thyroid and breast lesions in US images [2]. The accuracies of these methods were comparable to or even higher than those of radiologists. While the models perform well, explaining the decisions of CNN models and identifying which image regions contribute to these decisions remain an open and active area of research due to the “black box” decision-making nature of the models. Yet, the ability to understand how and why a classification model made a decision is essential for clinical diagnoses of cancer and a key requirement for trustworthy systems. These important concerns have motivated this study.

Several attempts have been made to explain how CNN models classify objects in natural images in general [8,9], and a few studies have investigated CNN decision explainability in breast US images in particular [10,11,12,13,14]. Although these efforts made serious attempts to examine the link between DCNN model decisions and regions of US images with the assistance of subject specialists, no effective visualization methods have been fully investigated to establish possible links from image texture features extracted by CNN with domain-known cancer characteristics, but identifying such links is highly desirable for building trusts in the model’s decisions.

This paper presents a new approach for explaining DCNN classification decisions regarding benign and malignant lesions by mapping saliency maps and cancer signs identified by clinicians. We hypothesize that analyzing the image regions used by the classification models with reference to domain-known breast lesion characteristics will lead to better explainability of the model’s decisions. We, therefore, propose a framework for lesion classification where establishing the mapping becomes an integral part of the proposed framework. We then conduct a thorough evaluation within the framework. The evaluation uses two datasets containing 1298 US images of breast lesions acquired from machines of different makes in two hospitals. The contributions of this study are as follows: (1) investigating the applicability and performance of EGrad-CAM and Ablation-CAM methods towards understanding CNN classification of breast lesions from US images; (2) proposing a new framework with mapping between the DCNN model decision and the known cancer characteristics such as calcification, echogenicity pattern, shape, and margins; (3) adapting two different DCNN networks and training models in the framework; and (4) collecting and evaluating evidence about the overall effectiveness of our methods through extensive evaluation of US images of breast lesions collected from different medical centers and annotated by experienced radiologists. This paper is the first attempt to directly link the saliency maps and cancer characteristics to explain DCNN decisions for breast lesion classification.

The remaining parts of the paper are organized as follows. Section 2 provides an overview of breast ultrasound image reporting systems and reviews the related literature. Section 3 presents our framework, the datasets, and the experiment setups. Section 4 provides the results of the evaluation of the proposed framework. Finally, several key issues are discussed in Section 5 before Section 6 concludes the paper.

## 2. Background and Related Work

This section provides an overview of the research background, followed by a discussion of related work. First, the known US characteristics or signs of breast lesions are summarized. These signs will support the DCNN decision analysis as described in Section 3. Second, we provide background information on the explainability of DCNN. Third, we present a literature review of breast lesion classification methods and the applications of explainability in DCNN using breast US images.

### 2.1. ACR BI-RADS Lesion US Image Descriptors

Due to intrinsic speckle noise, US images have low contrast between different types of tissue, and are of low image quality and hence difficult to read. Radiologists with varying experience may use different criteria to describe lesion malignancies. To reduce inter-observer variability, ACR BI-RADS defines a standard set of US image descriptors [4], which are briefly summarized as follows:

- Shape. The shape of a breast mass can be *oval* (or *elliptical*), *round*, or *irregular* (not oval nor round mass).
- Orientation. The lesion orientation refers to its long axis. If the long axis is parallel to the skin surface, the lesion is said to be *parallel*; otherwise, it is *anti-parallel*.
- Margin. Margin refers to the edge or border of a lesion. The margin of a lesion can be *circumscribed* when the margin of a lesion is *clearly* defined or *well separated* from the surrounding tissue, or *not circumscribed*.
- Echo Pattern. Mass echo pattern descriptors depend on tissue having different echotexture references such as *anechoic*, *hyperechoic*, *complex cystic*/*solid hypoechoic*, *isoechoic*, or *heterogeneous*.
- Posterior Feature. The posterior feature describes the attenuation properties of masses related to their acoustic transmission. The outcome can be *no feature*, *enhancement*, *shadow*, or *combined*.
- Calcification. Calcification is a tiny fleck of calcium usually visible as a bright spot, i.e., a small hyperechoic region inside the lesion in the US image. It is an important malignancy characteristic and should be further evaluated via biopsy.

### 2.2. Explainability of DCNN Model Decisions

DCNNs consist of numerous weight parameters, finely adjusted during training, making the resulting features—achieved through convolution, activation, and pooling—challenging to interpret. Recognizing this, significant progress has been made in developing visualization techniques to map the connection between specific input regions or pixels and DCNN predictions. There are two types of techniques: post hoc methods, such as vanilla gradients [15], Smoothgrad [16], CAM [9], and Grad-CAM [8], which highlight the significant input features that influence model decisions, and explainable-by-design approaches, which integrate explainability directly into the model’s architecture. These approaches either adopt simpler, inherently interpretable structures—like linear regression or decision trees [17]—or design more complex configurations, such as certain types of deep neural networks, to be transparent [18]. In this study, we mainly focus on post hoc methods and review some of the most promising ones.

LIME (Local Interpretable Model-Agnostic Explanations). LIME is a general-purpose explanation scheme for classifiers built with machine learning for natural images. By generating local explanations, LIME helps to identify the regions within an input image that contribute most significantly to the classification decisions [19]. Although LIME can be applied to explain cancer prediction models on US images, certain limitations should be acknowledged. The local explanations may not capture the global relationships and dependencies of certain cancer signs present in the image. The localized nature of LIME may also miss the broader context necessary to interpret lesion predictions correctly. The stability and consistency of LIME explanations may also vary, depending on the choice of hyperparameters and perturbation methods. Reliable explanations, therefore, require careful parameter tuning and robustness evaluations [19].

SHAP (SHapley Additive exPlanations). SHAP has attracted attention in explaining cancer prediction models based on ultrasound images. By assigning importance values to features, SHAP offers insights into the relative contributions of different visual attributes in ultrasound images to the model’s predictions. Such global and local explanations help elucidate individual features’ impact on the cancer prediction [20]. However, the computational complexity of SHAP can pose serious challenges, particularly with large-scale datasets and complex models. The interpretation of SHAP explanations also requires certain levels of expertise due to its reliance on game theory, limiting its accessibility to non-experts [20].

CAM (Class Activation Mapping). CAM is an approach that produces a heatmap showing how each input image pixel contributes to CNN classification decisions [9]. In the CAM methods, a GAP layer is required to visualize the weighted sum of the resulting feature maps at the pre-softmax layer. Grad-CAM, a gradient-weighted class activation mapping scheme, was developed to generalize CAM without requiring a GAP layer [8]. EGrad-CAM [21] further uses the entropy of feature maps as a measure to select and only visualize feature maps with a high amount of information. Another approach of utilizing the gradient-free methods to visualize CNN is introduced by Score-CAM [22], Ablation-CAM [23], and Clustered-CAM [24]. In this study, we investigate the effectiveness of EGrad-CAM and Ablation-CAM in visualizing classification decisions by CNN models for breast lesions.

The gradient of the score for class *c* (pre-softmax) $\alpha_{k}^{c}\;$ is computed by EGrad-CAM using Equation (1), where *k* is the index of the feature map at a spatial location (x, y).

(1) $$\alpha_{k}^{c}=\sum_{x,y}\frac{{\partial S}_{c}}{{\partial f}_{k}(x,y)}$$

(2) $$H=-\sum_{n=0}^{255}p_{n}\log_{2}p_{n}$$

EGrad-CAM uses the entropy of feature maps to measure the amount of information within each feature map and visualizes the most informative feature maps. The entropy is calculated using Equation (2), where $p_{n}$ represents the probability of value n appearing in the feature map.

Following that, a weighted sum of all these feature maps is calculated. Then, a ReLU function is applied to remove negative weights. Finally, heatmap scores are produced by aggregating feature maps with entropy greater than 0 or a predefined threshold. The final visualization output of EGrad-CAM is calculated using Equation (3).

(3) $$M^{EGrad-CAM}=\left\{\begin{array}{c} ReLU\;\left(\Sigma_{k}\alpha_{k}^{c}f_{k}\left(x,y\right)\right)\;H\geq threshold\;\\ Freeze\;FM\;otherwise \end{array}\right.$$

Ablation-CAM determines which feature map units are most important based on their class, using ablation analysis. The basic principle behind Ablation-CAM is to freeze (or remove) each feature map from the last convolution layer and then check whether the prediction class remains the same. The ablated or deleted feature map is not a characteristic feature when the classification decision is not changed. Feature maps’ significance value $\beta_{k}^{c}$ is the reduction in activation score (pre-softmax) when the feature map $F_{k}$ is deleted.

(4) $$\beta_{k}^{c}=\frac{S_{c}-S_{c}^{k}}{S_{c}}$$

where $S_{c}$ is the activation score of class *c*, and $S_{c}^{k}$ is the score for class *c* when feature map *k* is eliminated. In Ablation-CAM, activation maps and associated weights are combined linearly from Equation (4), and the heatmap visualization is generated using Equation (5) [23].

(5) $$M_{c}^{Ablation-CAM}=ReLU\left(\Sigma_{k}\beta_{k}^{c}F_{k}\right)$$

### 2.3. Lesion Classification from US Images

Many DCNN models have been developed to classify objects in natural images. It has been shown that VGG19 [25] and GoogleNet [26] among those DCNN models perform particularly well on the ImageNet dataset. VGG19 has 47 layers, 16 convolutional layers, 3 fully connected layers, and approximately 144 million weight parameters, whereas GoogleNet has 9 inception modules, is 22 layers deep (27 including the pooling layers), has a GAP layer at the end of the last inception module, and has approximately 7 million weight parameters. The use of DCNN architectures in the medical field has been investigated in several studies [27]. Large datasets are often required to train such complex DCNN architectures. Medical image datasets are often limited in size, and so transfer learning (e.g., [25,26]) has been utilized to overcome the limitation. Several studies have followed this approach for classifying breast lesions in US images and achieved high performance [2,10,28]. A generic VGG19-based DCNN architecture was adapted for classifying thyroid nodules and breast lesions from US images [2]. The breast lesion model, trained on 672 breast lesion images, achieved an accuracy of 89%. Therefore, we built breast lesion classification models based on the adapted VGG19 (BNet) and GoogleNet architectures for this study.

Neiman et al. [11] trained GoogleNet-based models using mammography and ultrasound images. The Grad-CAM method was used to gain insight into the decisions of lesion malignancy made by the models. Their study revealed that the models’ decisions over malignant lesions depend mainly on the boundaries of the lesions. Tanaka et al. [10] developed an ensemble DCNN model using VGG19 and Resnet152 over US images of 897 malignant and 639 benign cases. Using the deconvnet method, they visualized the important input image regions for the model decisions. They found that benign cases are more likely to be detected than malignant ones. In [29], the authors trained a Resnet18 network to classify breast lesions from US images. They applied Grad-CAM to locate the lesions and found that the main attention of their models focused on the lesion regions. In [12], a weakly-supervised deep learning algorithm was developed to diagnose breast cancer without requiring image annotation. A weakly-supervised algorithm was applied to VGG16, ResNet34, and GoogleNet. They applied the CAM method to locate breast masses. Recently, AlZoubi et al. [13] presented a comprehensive evaluation on transfer learning based solutions and automatically designed networks. The authors explored the use of saliency maps (EGrad-CAM and Ablation-CAM) to explain the classification decisions made by six DCNN models. The investigation showed that saliency maps can assist in comprehending the classification decisions.

## 3. Materials and Methods

### 3.1. Data Collection and Annotation

In this study, two datasets of ultrasound images of breast lesions were retrospectively collected by the sponsor of this research from Pudong New Area People’s Hospital and Renji Hospital in Shanghai, China. Both datasets were shared for analysis between January 2019 and July 2019. All breast grey-scale US examinations were performed in the hospital using US machines of different makes, including Siemens, Toshiba Apolio, GE Logic, and Philips.

The first dataset, Br-Dataset A, consists of 798 images of benign and 326 images of malignant lesions. Br-Dataset A is used to train models of different CNN architectures for lesion classification. The second dataset, Br-Dataset B, consists of 300 images of benign and 200 images of malignant lesions, and is used to analyze and explain CNN classification decisions via lesion characteristics. Each original ultrasound image contains a region showing one lesion in the breast mass. Three radiologists with more than ten years of experience identified the lesion region in the image and cropped the region manually by placing coordinate points on the lesion boundary using the software tool presented in [2]. The type of lesion (benign or malignant) in each region of interest (RoI) image was confirmed through histopathological assessment of tissue samples and served as the ground truth. In addition, all 500 images in Br-Dataset B were labeled by a radiologist with more than ten years of experience according to the ACR BI-RADS guidelines. Four lesion characteristics, i.e., shape, margin, echogenicity pattern, and calcification, were labeled and located using the software tool presented in [2]. More details of the annotation are given in Table 1.

**Figure 1:**
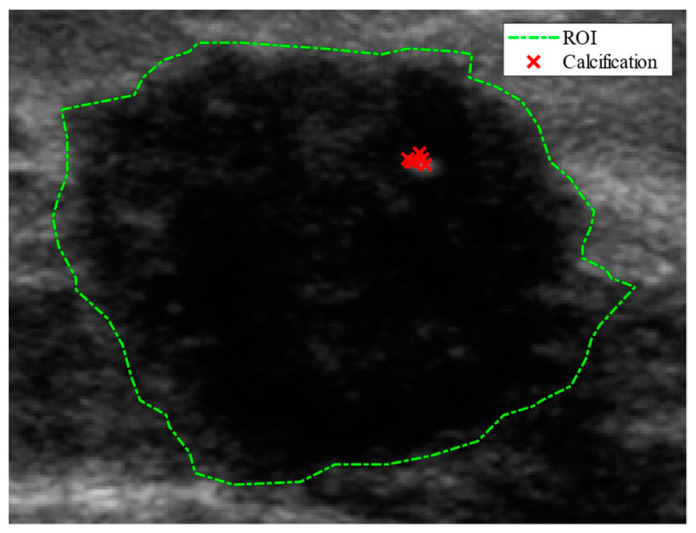
A malignant breast US Image with RoI lesion indicated in green, calcification points in red, irregular shape, and a non-circumscribed margin.

Figure 1 shows an example image of a malignant lesion with the lesion boundary annotated by the radiologist (green dash line). The lesion is considered as an irregular shape with a non-circumscribed margin, non-uniform echo, and calcification. It is worth mentioning that it is difficult for the radiologist to provide ground-truth annotations for echogenicity patterns at the pixel level. Instead, the radiologists only annotate the echogenicity pattern as *uniform* or *non-uniform* for the whole lesion image.

### 3.2. Methods

**Figure 2:**
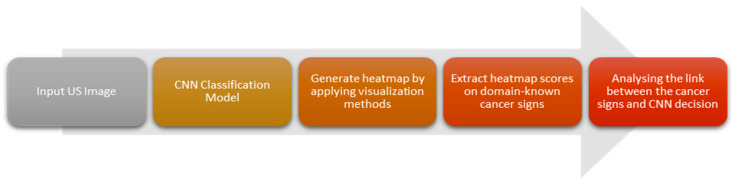
Our method of CNN decision understanding for breast lesion classification from ultrasound images.

Our proposed framework consists of five main consecutive steps: (1) input US image: preparing ultrasound images for classification and decision visualization; (2) CNN classification model: developing a CNN model for breast lesion classification; (3) heatmap generation: identifying the image regions contributing to the classification decisions using visualization techniques (e.g., EGrad-CAM); (4) extracting heatmap scores of image regions of domain known cancer characteristics; and (5) analyzing the connection between the visualization outputs and the significant lesion characteristics. Figure 2 illustrates the proposed framework.

It must be noted that the proposed framework is highly modular and general, and various methods can be used in each step of the process so long as the classification results are accurate and interpretable. In this study, we adapt the framework for breast lesion classification from US images. Each step has its selection of methods and is further explained in the following subsections.

#### 3.2.1. Ultrasound Image Preparations

This step refers to any necessary pre-processing operations before the input images are used for modeling. In this study, we undertake two tasks: RoI cropping and resizing. As explained in Section 3.1, RoI was cropped by three experienced radiologists using a free-hand cropping tool [2]. The cropping tool enables radiologists to upload a US image and performs three main functionalities: (1) identify the lesion boundary by locating the pixel positions of the border points; (2) identify the pixel locations of cancer characteristics, including calcification, echogenicity, shape, and margin; and (3) save the pixel locations of both lesion delineation and cancer characteristics with the corresponding lesion name (unique ID). Figure 1 shows an example of lesion boundary and calcification regions identified by the radiologists. Based on the lesion border points, a minimum-area rectangle was fixed around each lesion. The bounding box was expanded by an extra 8% margin to include the tumor microenvironment (TME) [30]. Since DCNN models work on fixed-size input images, the cropped RoI images were resized to the size required by the input layer of a specific network (e.g., 224 × 224 × 3) using bicubic interpolation. The pixel locations of cancer characteristics were used to including calcification, echogenicity, shape, and margin to assist in DCNN model decision explainability.

#### 3.2.2. DCNN Classification Model Development

At this step of the framework, DCNN models for breast lesion classification are developed. For this study, we constructed two alternative DCNN models, BNet and GNet, for comparison purposes.

BNet: Inspired by the work in [2], we developed a breast lesion classification model BNet with the same parameter setting as reported in [2] using Br-Dataset A. We trained a classification model using the DCNN architecture [25] pre-trained on the ImageNet dataset. To adopt the architecture for the intended purpose, we replaced the last fully-connected layer with a new fully-connected layer for binary classes and fine-tuned the softmax layer. Furthermore, the last ‘Dropout’ layer for BNet was set to 25%. Furthermore, we set the iteration number to 9080, the initial learning rate to 0.0001, and the mini-batch size to 8. Other parameters of the network remained unchanged [25].

GNet: We adapted a pre-trained DCNN model [26] that was originally trained on the ImageNet dataset. We replaced the last fully-connected and softmax layers with new layers that could be fine-tuned for our specific application. For binary classification, we added a new fully-connected layer to replace the last fully-connected layer of the original model. GNet was trained with the following parameters: 150 epochs representing 2250 iterations, the initial learning rate set to 0.0001, and a mini-batch size of 64. The remaining network parameters were set to their default values as specified in the original DCNN model [26].

#### 3.2.3. CNN Classification Decision Visualization

**Figure 3:**
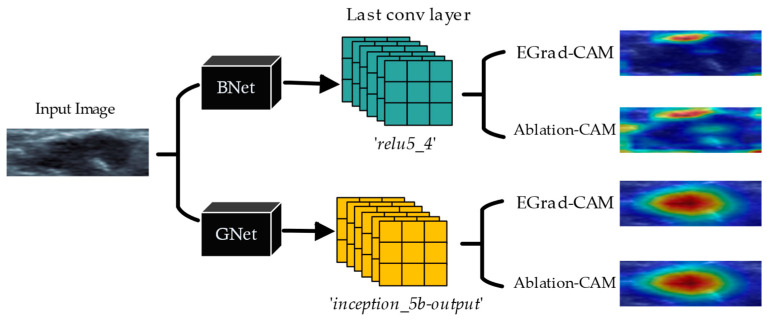
A visual representation of the CNN classification decision visualization using BNet and GNet models along with applied visualization techniques, i.e., EGrad-CAM and Ablation-CAM.

To gain insight into the classification decisions made by the DCNN models for distinguishing between benign and malignant breast lesions, we employ different visualization techniques: in this study, specifically EGrad-CAM [21] and Ablation-CAM [23]. By examining the generated heatmaps as described in Section 2.3, we gained insights into the importance of specific features or regions in the image that contribute to the overall classification decision made by the DCNN models. This study focuses on the last convolutional layers of BNet (‘relu5_4’ with size 14 × 14 × 512) and GNet (‘inception_5b-output’ with size 7 × 7 × 1024). Figure 3 illustrates an overview of the applying visualization techniques to the corresponding convolution layer of the BNet and GNet models.

EGrad-CAM: EGrad-CAM [21] calculates the Shannon entropy of each feature map in the desired layer and generates a heatmap based only on the feature maps with substantial information that significantly contributes towards the classification decision of BNet and GNet models. We applied Equations (2) and (3) to generate a heatmap for both the ‘relu5_4’ and ‘inception_5b-output’ layers of the BNet and GNet models, respectively.

Ablation-CAM: Ablation-CAM estimates the importance of each feature map by freezing the feature maps and comparing the model’s prediction class with and without the processed feature map using Equations (4) and (5). This process allows us to assess the impact of individual feature maps on the final classification decision. We also generate a heatmap for both convolutional layers of the BNet and GNet.

#### 3.2.4. Explaining CNN Classification Decision Using Lesion Characteristics

Although the visualization techniques provide valuable insights into the significance of individual pixels in the CNN model’s classification decisions, this information alone is inadequate for explaining the model’s decision without incorporating medical know-how about the lesion characteristics. This section proposes a new method to link the DCNN model decision heatmaps to the domain-known lesion characteristics.

Our method comprises two primary stages. Initially, we employ models and visualization techniques to approximate the significance of pixels in the RoI image lesion. Subsequently, we further scrutinize the heatmap scores of various cancer indications in the lesion image’s corresponding areas and their contributions to the CNN decision. By leveraging the ground-truth labels of the pixels or regions representing cancer characteristics, we analyze the heatmap scores of the corresponding characteristic locations for different prediction outcomes: True Negative (TN, i.e., correctly classified benign cases), True Positive (TP, i.e., correctly classified malignant cases), False Positive (FP, i.e., misclassified malignant cases), and False Negative (FN, i.e., misclassified benign cases). In essence, we explicate the visualization output by approximating the contribution of lesion characteristics such as calcification, echogenicity, shape, and margin to the classification of benign and malignant cases. Referring to US images with their corresponding cancer signs’ locations, we apply the following steps to determine the contribution of each cancer sign:

Step 1. Classify Images: Feed images to BNet or GNet to classify the images;
Step 2. Visualize CNN Decisions: Employ a visualization technique to generate a heatmap for the predicted class;
Step 3. Extract Heatmap Score: Extract the heatmap scores of the locations for the known cancer signs in the RoI image according to the ground truth;
Step 4. Calculate Ratios of Significant Scores: Determine the ratio of heatmap scores that are greater than or equal to 0.5 to quantify how much of the existing cancer sign was considered significant by the CNN in each image as follows:
  (6) $${Rs}_{i}=\left|\left\{p\;\right|p\in \;R_{i}\;and\;Hs\left(p\right)\geq 0.5\right\}|/|R_{i}|$$
  where *Hs*(*p*) stands for the heatmap score for pixel *p* within the region *R_i_*. The significance threshold for heatmap scores is set at 0.5, as it represents a balanced value within the normalized range of scores from 0 to 1. This choice was supported by our empirical findings, which demonstrated its efficacy in capturing substantial contributions within the heatmaps. This threshold effectively distinguishes between areas of high and low relevance to the model’s decision-making process, facilitating a more targeted analysis of cancer sign contributions.
Step 5. Aggregate Ratios Across Images: Calculate the average ratios for each sign across all correctly classified and misclassified cases (TN, TP, FP, FN) using Equation (7) to evaluate consistency in how the CNN considers cancerous signs across cases.
  (7) $${Av}_{Rs}=\left(\sum_{i=1}^{K}{Rs}_{i}\right)/K$$
  where K is the number of correctly or misclassified cases of a cancer type (benign or malignant).
Step 6. Categorize the Average Ratios: Based on the aggregated average ratios, categorize the contribution of each cancer sign into predefined levels, *No, Very Low, Low, Medium, and High,* using Equation (8).
  (8) $$Cc=\left\{\begin{array}{cc} \begin{array}{c} No\\ \begin{array}{c} Very\;Low\\ Low\\ \begin{array}{c} Medium\\ High \end{array} \end{array} \end{array} & \begin{array}{c} {Av}_{Rs}=0\\ \begin{array}{c} 0<{Av}_{Rs}\leq 0.2\\ 0.2<{Av}_{Rs}\leq 0.35\\ \begin{array}{c} 0.35<{Av}_{Rs}<0.5\\ \geq 0.5 \end{array} \end{array} \end{array} \end{array}\right.$$
  where the upper and lower bounds of the ${Av}_{Rs}$ ranges are empirically determined.

Our proposed solution (Steps 1 to 6) for estimating the contribution of each cancer sign in the final decision of CNN classification is represented in pseudocode in Algorithm 1.


**Algorithm 1: Explaining CNN Classification Decision Using Lesion Characteristics**


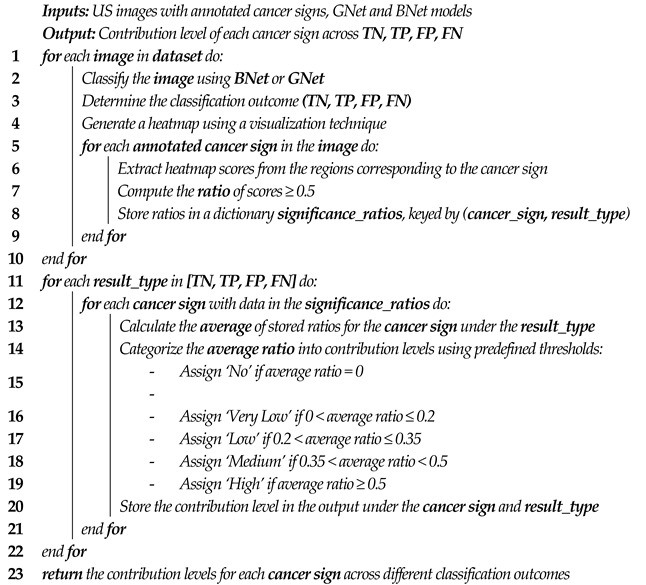



#### 3.2.5. Breast Cancer Characteristics to Assist in Model Decision Explainability

Using the ground-truth labels from Br-Dataset B, we examine the relationship between the classification models and the breast lesion characteristics as follows.

**Figure 4:**
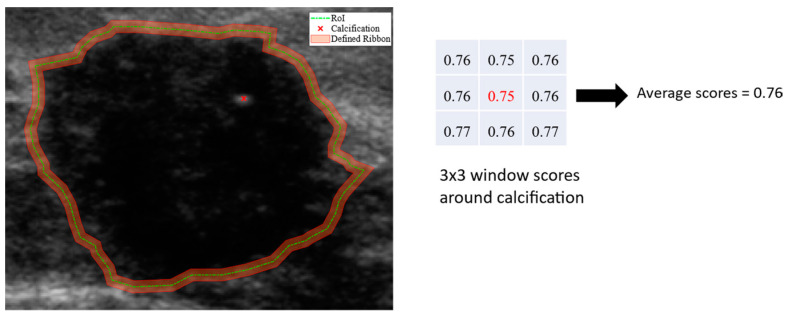
A US breast image with calcification point, uniformity pattern, and defined ribbon for margin and shape analyses. The table on the right displays a 3 × 3 window of calcification region (denoted in red), with the average score calculated using the method described earlier.

Calcification. We analyze the heatmap scores of all calcification regions present in both benign and malignant lesions in Br-Dataset B. To ensure sufficient coverage of the calcification region, we assess a 3 × 3 window around each calcification pixel, as depicted in Figure 4. By calculating the average importance score of these pixels, we obtain an indication of their contributions to the final decision made by the classification models. Finally, we categorize the contribution of calcification characteristics for correctly classified and misclassified cases across the entire dataset using Equations (7) and (8). This investigation involved using 76 images from the Br-Dataset B that exhibited the calcification cancer sign (comprising of 7 benign and 69 malignant images). The importance scores of 1111 calcification regions with 1025 in malignant cases and 86 in benign cases were evaluated in this study. Echogenicity Pattern. Based on the ground-truth in Br-Dataset B, all pixels within the lesion boundary indicate whether the echogenicity pattern of the lesion is uniform or non-uniform. To assess the contribution of this characteristic in the CNN model’s decisions, we used the methods described in Equations (7) and (8). From Br-Dataset B, a total of 40 cases with uniform patterns (34 benign and 6 malignant images) were compared to 460 cases with non-uniform patterns (266 benign and 194 malignant images).

Shape and Margin. To examine the influence of shape and margin on the classification decision of a model, we generated a ribbon around the lesion boundary provided by the ground truth. The ribbon was created by first generating a rectangle with a minimum area of the boundary points of the lesion. The width of the ribbon was then calculated using the average of the width and height of this rectangle (${AR}_{wh}$). Inner and outer boundaries were generated with the original lesion boundary in the center. The width of both boundaries was 2% of the ribbon width. This threshold was experimentally determined to ensure that there were enough pixels to analyze the shape and margin and to overcome the issue of frequent small sharp angles on the boundary. The resulting ribbon captured the pixels surrounding the original lesion boundary. The ratio of heatmap scores of the pixels within the defined ribbon region was computed, and the contribution of the margin and shape signs in the lesion classification decision was categorized using Equations (7) and (8). A malignant lesion with an irregular shape and non-circumscribed margins signs is illustrated in Figure 4. We compared a total of 445 cases of irregular shapes (245 benign and 200 malignant) with 55 cases of regular shapes (all benign) from Br-Dataset B. We also compared 166 cases of circumscribed margins (161 benign and 5 malignant) with 334 cases of uncircumscribed margins (139 benign and 195 malignant) from Br-Dataset B.

## 4. Experiments and Results

### 4.1. Experiment Setup

Several experiments were conducted to evaluate the effectiveness of different methods in explaining the decisions made by deep convolutional neural networks (DCNNs). The first experiment demonstrates and compares the performance of two classification models: BNet and GNet. The second experiment involves analyzing the image regions that contribute to the models’ decisions across groups of accurately classified lesions. The third experiment evaluates and compares two visualization techniques, EGrad-CAM and Ablation-CAM. The fourth experiment examines the use of characteristics of breast lesion cancer domains to explain both the BNet and GNet models. Finally, the significance of these characteristics is further analyzed by studying the links between CNN model decisions to the cancer characteristics. All the experiments were conducted on a machine with the following specifications: an Intel Xeon(R) W-2102 CPU @ 2.90 GHz and 16.0 GB RAM.

### 4.2. Training CNN Models

A stratified 10-fold cross-validation process was followed using *Br-Dataset A* to determine the classification accuracy of DCNN models. At each iteration, 10% of the lesion images were reserved for testing, while the remaining 90% were used for model training. This evaluation protocol was adopted to ensure a reliable accuracy estimate and a relatively small variance. The performance of the models was assessed with overall accuracy, true negative rate (TNR), and true positive rate (TPR). Table 2 first presents the average performances from the 10-fold cross validation (CV) on Br-Dataset A. The table then presents the test results of the best-performing BNet and GNet models selected from the cross-validation and the test results of the selected models on Br-Dataset B. The best-performing BNet and GNet models were selected according to the highest overall accuracy and the least difference between TPR and TNR.

### 4.3. Aggregated Heatmap Scores

**Figure 5:**
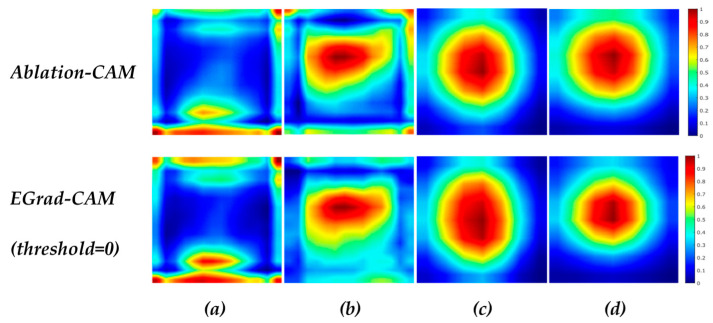
Average heatmap scores of (**a**) TN cases by BNet; (**b**) TP cases by BNet; (**c**) TN cases by GNet; (**d**) TP cases by GNet.

The first step towards explaining DCNN model decisions is to analyze the image regions that the models use to make correct predictions; we want to establish whether the DCNN model uses intranodular and peripheral regions to correctly classify the lesion. We analyzed the selected BNet and GNet models using Ablation-CAM and EGrad-CAM (threshold = 0) methods on Br-Dataset B. The goal is to understand the inter-class differences between malignant and benign cases as well as the intra-class similarities for each class. We used the heatmaps generated from the ‘inception_5b-output’ layer of GNet and the ‘relu5_4’ layer of BNet, aggregating the heatmaps created from all correctly classified RoI images into a single average heatmap. These heatmaps represent the important regions for TP and TN cases. Figure 5 shows the aggregated heatmaps. 

In Figure 5, a pixel with a value of 0 (blue color) indicates no contribution to the classification decision, while a value of 1 (red color) signifies a high contribution. Figure 5 reveals differences in the locations of important areas between benign and malignant lesions. For benign cases, BNet’s correct decisions are more influenced by textures of lesion boundary (peripheral) regions, particularly in the bottom of RoIs (posterior regions), whereas textures in the middle and upper regions (mainly intranodular regions) play a more crucial role for malignant cases. In contrast, the GNet model bases its correct decisions mainly on the central areas (intranodular regions) for both malignant and benign lesions across both visualization methods. In particular, the most crucial areas for benign lesions are situated in the middle and downward parts, whereas for malignant lesions the highest scores are concentrated in the middle and upward areas of the lesions. It is clear that the BNet and GNet models use different image features and regions to predict lesion types due to the differences in their architectures and feature extraction.

In addition, for BNet, EGrad-CAM heatmaps show that in benign cases, approximately 60% of feature maps are utilized, while in malignant case, it is close to 36%, suggesting that a significant portion of the feature maps in the ‘relu5_4’ layer of BNet contribute little to its decisions. Similarly, for GNet, EGrad-CAM employs about 66% of feature maps for benign cases and 71% for malignant cases, indicating that many feature maps in the ‘inception_5b-output’ layer of GNet have little impact on GNet decisions.

### 4.4. Linking BNet and GNet Decisions with Domain-Known Cancer Characteristics

This section particularly examines the connection between the visualization of the decisions made by the BNet and GNet models and the domain-known lesion characteristics using Br-Dataset B. We explore the significant links between highlighted image regions in heatmaps and signs such as calcification, echogenicity pattern uniformity, shape, and margin using the methods described in Section 3.2.4 and Section 3.2.5.

#### 4.4.1. Decisions and Image Regions for Calcification

A comparison between the heatmap scores for calcification regions produced using by the Ablation-CAM and EGrad-CAM methods and classification results (TP, TN, FN, and FP) is presented in Table 3. The table shows the proportion of the pixels within the calcification regions with heatmap scores greater than or equal to 0.5, the threshold for the ratio of importance (see Equation (8)).

For the BNet model, the Ablation-CAM indicates that the calcification sign made a medium contribution (38%) towards TP predictions, a very low contribution (16%) to TN predictions, no contribution to FN, and low contribution (31%) to FP cases. The EGrad-CAM top 95% method also indicates that the calcification sign contribution for TP predictions was 20%. For TN and FN cases, the contributions were none. The contribution of the calcification sign towards FP cases is 6%. Although much lower in percentage, these readings are consistent with those from the Ablation-CAM. The EGrad-CAM top 95% method also finds that an average of 72% of feature maps had no contribution to the calcification scores.

For the GNet model, the table shows that using Ablation-CAM, a large proportion of calcification pixels had high heatmap scores at or above the threshold for all cases, particularly for the FP cases, where all pixels in the calcification region had a high heatmap score. These findings show the significant role played by calcification regions in the decision-making process of the GNet model. Furthermore, the table reveals that calcification pixels play a more significant role in classifying malignant cases than the benign cases, highlighting the importance of the calcification regions in predicting malignancy. The EGrad-CAM top 95% method reveals a consistent pattern, but the calcification pixels contribute less to the FP errors than that indicated by Ablation-CAM. The EGrad-CAM top 95% also finds that, on average, 33% of feature maps make no contributions to the calcification scores.

**Figure 6:**
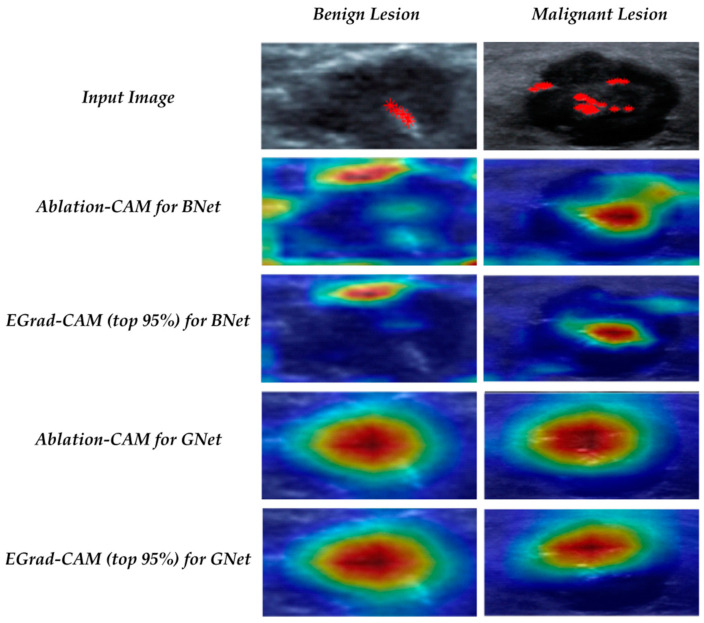
Visualization of correctly classified benign and malignant lesions with calcification.

Figure 6 shows some example heatmaps for a benign case and a malignant case correctly classified with the BNet and GNet models. The top row shows the calcification ground-truth marked in red crosses on the input images. The figure illustrates that calcification regions are crucial in predicting malignancy by both BNet and GNet models shown by both visualization methods. For the benign case, calcification regions have lower importance than the upper margin regions in the BNet’s decision, but still play a role in the GNet’s decision.

#### 4.4.2. Decisions and Image Regions for Echogenicity Patterns

We analyzed the significance of the echogenicity uniformity for benign and malignant lesions using the BNet and the GNet models. Table 4 presents the proportion of the pixels within the uniformity and non-uniformity regions with heatmap scores at or above the threshold. For BNet, the EGrad-CAM (top 95%) scores in the table reveal similar patterns for both uniform and not uniform scenarios: 6% and 8% in TN, 12% and 22% in TP, 11% and 18% in FP, and 2% and none for FN. These findings suggest that the non-uniform echogenicity pattern has a very low contribution towards BNet’s decisions across predicted outcomes, while the uniform pattern has a very low contribution to TN and FP predictions and a still low, but relatively higher, influence on TP cases.

The Ablation-CAM heatmap analysis reveals that the non-uniform echogenicity pattern has a very low contribution to the BNet decisions in TN, FP, and FN cases, while it has a still low but relatively higher contribution in TP cases. For images with uniform patterns, the contribution to the BNet decisions is very low for TN cases and low for TP and FP cases. It should be noted that the BNet model classified all malignant breast lesions with uniform patterns correctly. Table 4 also shows that the contribution of uniformity and non-uniformity patterns to the decisions made by GNet is high for TN, TP, and FN predictions, while it is medium for FP cases. Although the ratios highlighted by the EGrad-CAM top 95% are lower than those by Ablation-CAM, the pattern across the various cases is consistent to that revealed by Ablation-CAM.

EGrad-CAM with top 95% entropy values reveal that an average of 62% and 68% of feature maps of BNet have no contributions to the uniformity and non-uniformity scores, respectively. For the GNet model, averages of 35% and 36% of feature maps had no contributions to the uniformity and the non-uniformity scores, respectively.

**Figure 7:**
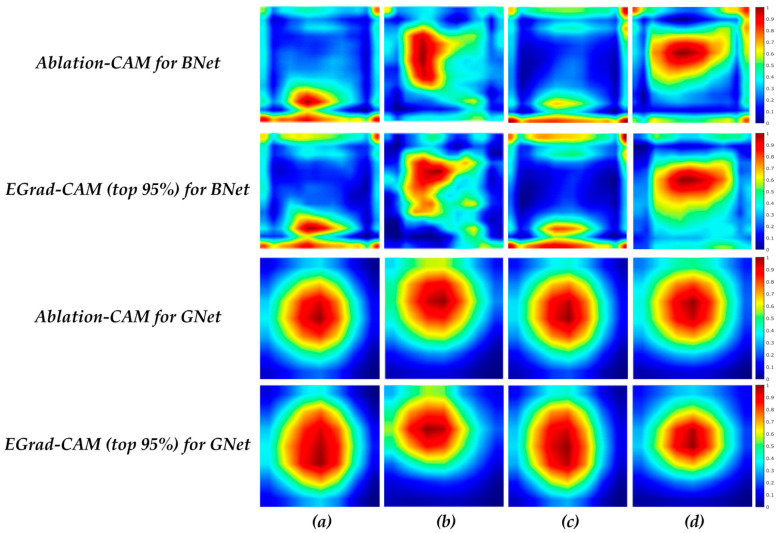
Average heatmap scores of (**a**) TN cases with uniformity; (**b**) TP cases with uniformity; (**c**) TN cases with non-uniformity; and (**d**) TP cases with non-uniformity.

Figure 7 illustrates the average heatmaps for all images in Br-Dataset B with uniformity and non-uniformity patterns for correct predictions. For correct benign predictions, regardless of the echogenicity patterns, the BNet model tends to focus more on the boundary areas of lesions. For correct malignant predictions, the pixels inside the lesion play a more significant role in the BNet decisions. When comparing TP cases with uniform and non-uniform patterns, some differences can be observed in the heatmaps. For uniform lesions, the scores are distributed vertically on the left side of the images, leaning towards the middle. On the other hand, for non-uniform cases, the scores are concentrated in the core and upper half of the images. In comparison with BNet, GNet again tends to focus on the center regions of the lesions for both echogenicity patterns, which are similar, like the case for BNet. When comparing TP cases with uniformity versus non-uniformity patterns, we find that uniform cases exhibit a distribution that is more inclined towards the left corner of the lesions. In contrast, for non-uniform cases, the distribution of scores is more concentrated around the center of the lesions. It is worth noting that the boundary of the lesion contributes much less to the decisions made by GNet than those by BNet.

#### 4.4.3. Decisions and Image Regions for Shapes

Table 5 presents the ratios of the pixels in the defined boundary ribbons of regular and irregular shaped lesions with the heatmap scores at or above the threshold. For BNet decisions, both visualization methods revealed very low ratios for all classified cases of both shapes. The finding supports the conclusion that the lesion shape has a very low contribution to the decisions made by BNet. For GNet decisions, the visualization methods show low to very low contributions by both shape patterns, but the contributions of this cancer sign to GNet decisions are higher than those for the BNet decisions.

It is also worth noting that the EGrad-CAM top 95% has found that, on average, 69% and 61% of feature maps make no contributions to the irregular and regular shapes of the lesions correctly classified by BNet, respectively, and that 36% and 38% of feature maps have no contribution to the irregular and regular shapes of the lesions classified by GNet, respectively.

**Figure 8:**
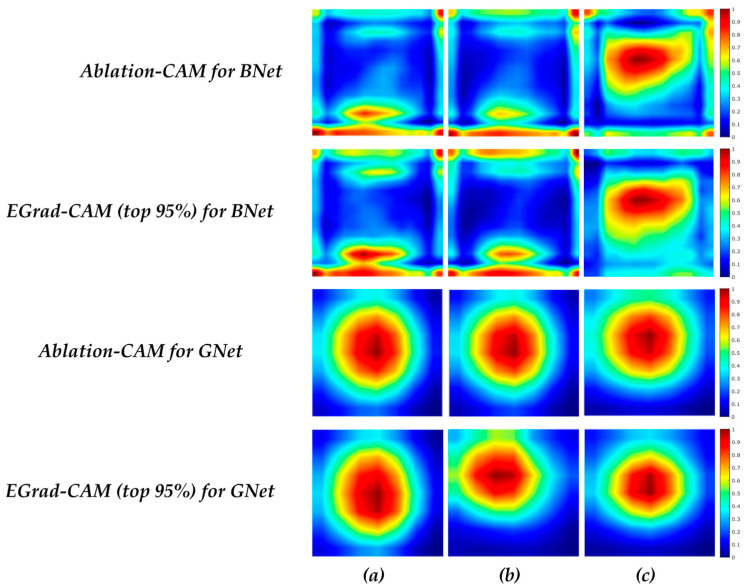
Average heatmap scores of (**a**) TN cases with regularity; (**b**) TN cases with irregularity; and (**c**) TP cases with irregularity.

Figure 8 depicts the average heatmap scores of all correctly classified images in the Br-Dataset B, with regular and irregular shapes obtained from the BNet and GNet models. For the TN cases with irregular shapes, BNet again focuses on the boundary of lesion. On the other hand, in TP cases with irregular shapes, the pixels inside the lesion play a more significant role in BNet’s decisions. It can be observed that the heatmap score distributions of the TN cases with irregular and regular shapes are similar, but for the cases with regular shapes, the bottom parts of the lesions contribute more to BNet’s decisions than the cases with irregular shapes. As for GNet, for the TN cases with both regular and irregular shapes, the distribution of heatmap scores is similar, indicating that GNet tends to focus on the centers of the lesions regardless of the shape. For the TP cases with irregular shapes, GNet assigns higher importance and concentrates on the upper half of the lesions, suggesting that this region plays a significant role in the correct classification.

#### 4.4.4. Decisions and Image Regions for Margins

Table 6 presents the ratios of the pixels within the defined ribbon regions for circumscribed and not circumscribed margins of the lesions with the heatmap scores at or above the threshold for both the BNet and GNet models. For BNet, both visualization methods show that circumscribed or non-circumscribed margins make very low contributions toward all classified cases. For GNet, the visualization methods show that both circumscribed and non-circumscribed margins have a greater impact on all possible classification outcomes than BNet does. The margin characteristics also have an impact on misclassified cases, indicating that this specific type of characteristics cannot play a good role in separating benign and malignant lesions for either the BNet or GNet models. It is worth noting that the EGrad-CAM top 95% method again reveals that, on average, 62% and 71% of feature maps showed no contributions to circumscribed and non-circumscribed margins of the lesion in cases correctly classified by BNet, respectively. The same method also reveals that, on average, 37% and 36% of feature maps had no contribution to the circumscribed and uncircumscribed margin scores in cases correctly classified by GNet, respectively.

**Figure 9:**
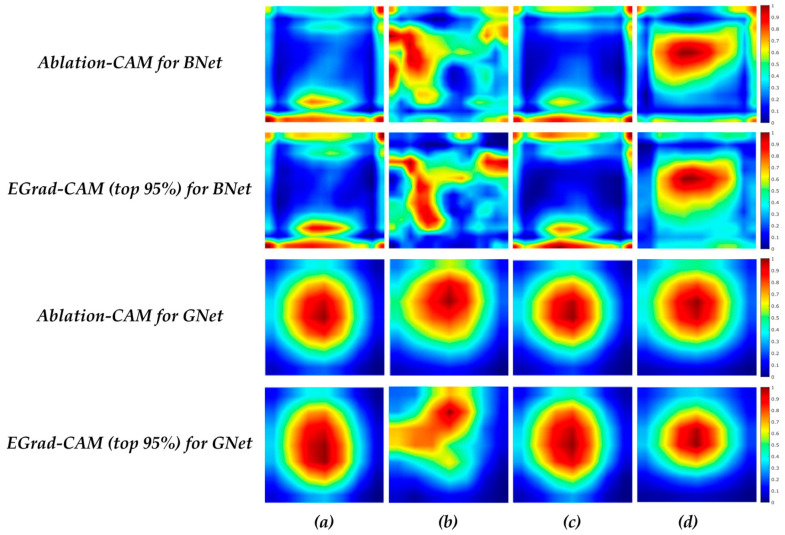
Average heatmap scores of (**a**) TN cases with circumscribed; (**b**) TP cases with circumscribed; and (**c**) TN cases with not circumscribed; and (**d**) TP cases with not circumscribed.

Figure 9 illustrates the average heatmaps of the correctly classified images by the BNet and GNet models, considering circumscribed and non-circumscribed margins. For TN cases, regardless of margin types, the BNet model emphasizes on the boundaries of the lesions. In particular, the BNet model assigns higher importance to the bottom boundaries of the lesion for the cases with circumscribed margins than those with non-circumscribed margins. The upper boundaries contribute more to the BNet decisions for the cases with non-circumscribed margins. For TP cases, the textures within the lesions influence the BNet’s decisions more. In particular, the heatmap scores of lesions for the cases with circumscribed margins are vertically distributed in the left half of the images. In addition, the textures on the top right side of the images also strongly influence the BNet decisions. On contrast, the heatmap scores for the cases with non-circumscribed margins are horizontally distributed and located in the upper half of the lesions. The results obtained from EGrad-CAM (top 95%) for TN and TP cases are similar to those of Ablation-CAM. The figure also shows that the distribution of heatmap scores for TN cases with both circumscribed and non-circumscribed margins is similar for the GNet model, focusing on the central regions of the lesions. Regarding the cases with circumscribed margins, Ablation-CAM assigns higher importance to the pixels within the lesions. At the same time, EGrad-CAM (top 95%) shows a distribution of heatmap scores that emphasizes upward textures and leans towards the left textures of the images.

In summary, the BNet model assigns importance of varying degrees to different known cancer characteristics, leading to differences in their contributions to the final decisions. The BNet model considers the lesion boundary as a crucial area for TN cases while focusing on the lesions’ internal textures for TP cases. The presence of calcification is a reliable factor for distinguishing malignant from benign lesions. The contribution of uniform patterns is slightly higher in TP cases than in TN cases. In contrast, the contribution of non-uniform patterns is more significant in TP cases than in TN cases. The contribution of irregular and regular shapes in TN and TP cases is similar. Furthermore, regardless of their margin type (circumscribed or not circumscribed), TN and TP cases contribute similarly to the BNet model decisions. As for the GNet model, it can be observed that different cancer sign characteristics have varying contributions to the GNet decisions. Overall, the GNet model places greater importance on the core of the lesion in both TN and TP cases. Calcification is a distinctive sign that the GNet model effectively utilizes to differentiate between benign and malignant lesions. The contribution of the non-uniform characteristic is slightly higher in TN cases than in TP cases. Conversely, a uniform pattern contributes more to TN cases than TP cases. The impact of irregular and regular shapes on TN and TP cases is similar, with no significant difference in their contributions. Similarly, the contribution of circumscribed and non-circumscribed margins to the GNet’s final decision is not much different for TN and TP cases.

### 4.5. Ranking Cancer Characteristics Importance in GNet and BNet Decisions

**Figure 10:**
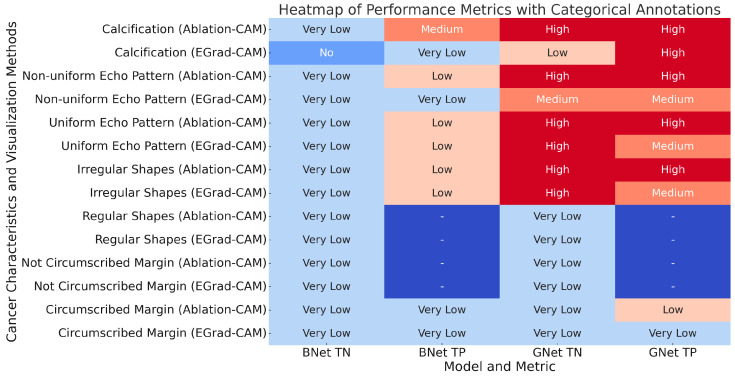
Cancer characteristic contributions for BNet and GNet with two visualization methods.

After comparing the results using two visualization methods on the BNet and GNet models, we now summarize and compare the contributions of different known cancer characteristics to the BNet and GNet model decisions. The contributions are converted into categorical values according to Equation (8). Figure 10 summarizes the findings for correctly classified cases.

Calcification: Both BNet and GNet models utilize calcification in their classification decisions, with a greater importance observed for TP cases. This characteristic can be used to explain the model’s decisions regarding malignant lesions, establishing a direct link between the CNN models and this lesion characteristic.

Uniformity Pattern: Figure 10 reveals that the contribution of non-uniformity to BNet’s decisions for correctly classified cases is lower than those of GNet. The contribution remains the same across the two visualization techniques for the TN cases, while for the TP cases, only Ablation-CAM shows a higher contribution for BNet. On the other hand, the contribution of non-uniformity to the GNet’s decisions for both TN and TP cases is similar across the two visualization techniques. The contribution of uniformity to BNet’s decisions for both TN and TP cases is also lower than those of GNet. As for the uniformity, the contribution remains the same for the TN cases across the visualization techniques, and the same pattern is observed for the TP cases. Furthermore, the contribution of uniformity to GNet’s decisions is higher for TN cases than TP cases. These variations in the uniformity feature across different CNN models may be attributed to the distribution of this feature within the entire lesion.

Shape: Figure 10 shows that the contribution of irregular shapes to the decisions by BNet for both TN and TP cases is similar to that of GNet, except for Ablation-CAM, which assigns a higher weight to irregular shapes for GNet. As for the regular shapes of lesions, the figure shows that the contribution to BNet’s decision is the same as that of GNet for both TN and TP cases. Overall, both regular and irregular lesion boundary regions have very low contributions to the decisions of benign and malignant lesions. However, when combined with other signs, they can help to explain the classifications of benign and malignant lesions.

Margin: Figure 10 shows that the contribution of circumscribed margins to the final decision of BNet is similar in both TN and TP cases across different visualization techniques. In the case of GNet, EGrad-CAM shows higher contributions compared to the former Ablation-CAM. The contribution of non-circumscribed margins to the decisions of BNet is the same as those by GNet across the visualization techniques. Similar to the shape characteristic, both circumscribed and non-circumscribed image lesion boundary regions have very low contributions to benign and malignant classification decisions. However, they can still be used in conjunction with other signs to explain the classifications of benign and malignant lesions.

## 5. Discussions

**Figure 11:**
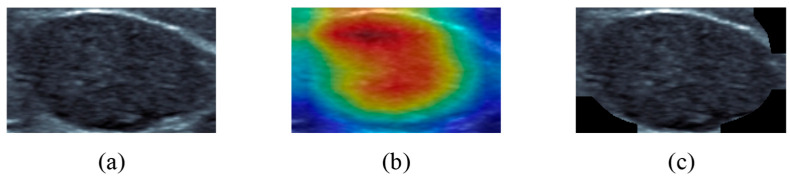
(**a**) RoI US image of a benign lesion; (**b**) Ablation-CAM heatmap of BNet; (**c**) corresponding explanation map.

In this study, we adapt and evaluate two CNN visualization techniques, EGrad-CAM and Ablation-CAM. In this section, we further discuss the performance of these two algorithms in identifying image regions that contribute to classification decisions. Our study adopted the approach of obtaining the ground-truth reference from human experts. However, this method can be time-consuming and subjective, relying on the observer’s opinion and experience in image reading. Alternatively, quantitative methods using explanation maps have been proposed to evaluate the reliability of generated heatmaps [23]. Explanation maps are created using a visualization technique, enhancing the subregions of the original image. For our experiment, we selected the top 80% of pixels from the localization map (i.e., heatmap). By multiplying this modified localization map with the original image, we generate an explanation map for the image. Figure 11 shows an example of the explanation map using the Ablation-CAM technique and the top 80% of pixels.

For the purpose of evaluating different CNN visualization techniques, we separately used the original test images and their explanation maps as inputs to feed into a given DCNN model for making classification decisions. We then measured three performance metrics: the average drop in prediction confidence, the percent (%) increase in prediction confidence, and the percent (%) win in confidence [23]. The average drop % in confidence measures the percentage decrease in the model’s prediction scores when an explanation map is given as input instead of the original image. The calculation is based on the confidence score ($Y_{i}^{c}$) of the original image and the explanation score ($O_{i}^{c}$) of the explanation map. The *max* function is used to exclude the images with $O_{i}^{c}>Y_{i}^{c}$. The metric is expressed as follows:

(9) $$Average\;Drop\;\%=\frac{1}{N}\sum_{i=1}^{N}\frac{\max\left(0,Y_{i}^{c}-O_{i}^{c}\right)}{Y_{i}^{c}}\;*\;100$$

The percent increase in confidence measures the rate at which the output scores of the model increase when only an explanation map is provided as input. It indicates how well the explanation map enhances the model’s confidence. The metric is calculated as follows:

(10) $$Percent\;increase\;in\;confidence=\sum_{i=1}^{N}\left(\frac{1_{Y_{i}^{c}<O_{i}^{c}}}{N}\right)\;*\;100$$

where $1_{Y_{i}^{c}}\;$ returns 1 if the argument is true. In both equations, N represents the number of images in the test dataset. An additional measure, the “Win%” metric, determines the percentage of instances where one visualization technique outperforms another by reducing the model’s output score to a greater extent. This analysis considers only positive drop values, disregarding negative values.

To evaluate, we used the Br-Dataset B dataset as an unseen dataset and set the threshold for generating an explanation map to 80%. Table 7 summarizes the performance metrics.

It can be observed that Ablation-CAM outperformed EGrad-CAM for BNet in terms of the average percent drop in confidence and percent increase in confidence, but not in win%. For GNet, Ablation-CAM performed better than EGrad-CAM in the percent increase in confidence and win%, but EGrad-CAM was marginally better than Ablation-CAM for the average percent drop in confidence.

## 6. Conclusions

In this paper, we presented a new framework for explaining DCNN classification decisions of breast lesions in ultrasound images using saliency maps linking the DCNN decisions to known cancer characteristics from the medical domain. The first part of our work used transfer learning to build two DCNN classification models (BNet and GNet) that have performed very well in classifying benign and malignant lesions. EGrad-CAM and Ablation-CAM techniques were employed to visualize the important regions in breast lesions that influenced CNN’s decision. The methods produced different visualizations for distinguishing between benign and malignant tumors. The second part of the work focused on establishing a connection between the features extracted by the trained CNN and domain-known characteristics, including echogenicity, calcification, shape, and margin. The test results of our framework show direct links between the CNN model decisions and the domain-known characteristics that contribute differently to the model’s decisions. This observation of the CNN decision behavior corresponds with medical knowledge, such as the presence of calcifications, which often serve as a crucial indicator of malignancy.

Motivated by our findings, we intend to expand our research in multiple directions. First, while the BNet and GNet models achieved similar classification accuracies, we plan to explain the differences in the learnable features of both models. Second, we will further investigate why EGrad-CAM and Ablation-CAM produce different visualization outputs while testing the same model and images. Finally, we want to investigate the performance of our framework using different cancer types, such as thyroid with TI-RADS, and perhaps even other image modalities.

## Figures and Tables

**Table 1:** Lesion characteristics labeling details.

Lesion Characteristic	Number of Samples
Shape	55 images of benign lesions were labeled as *regular* (elliptical or round), and 450 images of 245 benign and 200 malignant lesions were labeled as *irregular*.
Margin	166 images (161 benign and 5 malignant) were labeled as *circumscribed*, and 334 images (139 benign and 195 malignant) were labeled as *not circumscribed*.
Echogenicity pattern	460 images of 266 benign and 194 malignant lesions were labeled as *non-uniform*, while 40 images of 34 benign and 6 malignant lesions were labeled as *uniform*.
Calcification	76 images of 69 malignant and 7 benign lesions were found with calcification. The radiologist manually located 1025 regions of calcification in malignant lesions and 86 regions of calcification in benign lesions.

**Table 2:** Classification performance of BNet and GNet models.

Models	Br-Dataset A (CV Average)			Br-Dataset A (CV Best)			Br-Dataset B (Best)		
	Accuracy	TNR	TPR	Accuracy	TNR	TPR	Accuracy	TNR	TPR
BNet GNet	88.2%	92.6%	77.4%	91.1%	88.8%	96.9%	86.8%	84.0%	91.5%
	89.4%	86.9%	81.3%	87.5%	88.4%	84.6%	84.0%	79.0%	92.0%

**Table 3:** BNet and GNet heatmap scores for calcification characteristics of TN, TP, FP, and FN cases.

Heatmap Score Range ≥ 0.5	BNet				GNet			
	TN	TP	FP	FN	TN	TP	FP	FN
Ablation-CAM	0.16	0.38	0.31	0.00	0.67	0.88	1.00	0.83
EGrad-CAM (top 95%)	0.00	0.20	0.06	0.00	0.32	0.71	0.64	0.47

**Table 4:** Heatmap scores of echogenicity pattern uniformity on TN, TP, FP, and FN cases.

Heatmap Score Range ≥ 0.5	BNet								GNet							
	Not Uniform				Uniform				Not Uniform				Uniform			
	TN	TP	FP	FN	TN	TP	FP	FN	TN	TP	FP	FN	TN	TP	FP	FN
Ablation-CAM	0.08	0.21	0.15	0.04	0.13	0.28	0.32	-	0.56	0.58	0.49	0.60	0.57	0.56	0.45	0.59
EGrad-CAM (top 95%)	0.06	0.12	0.11	0.02	0.08	0.22	0.18	-	0.48	0.43	0.30	0.32	0.53	0.36	0.31	0.50

**Table 5:** Heatmap scores of lesion shape characteristics on TN, TP, FP, and FN cases.

Heatmap Score Range ≥ 0.5	BNet								GNet							
	Irregular				Regular				Irregular				Regular			
	TN	TP	FP	FN	TN	TP	FP	FN	TN	TP	FP	FN	TN	TP	FP	FN
Ablation-CAM	0.10	0.13	0.13	0.09	0.12	-	0.13	-	0.17	0.29	0.19	0.30	0.16	-	0.26	-
EGrad-CAM (top 95%)	0.08	0.08	0.08	0.06	0.08	-	0.09	-	0.16	0.17	0.13	0.19	0.15	-	0.09	-

**Table 6:** Heatmap scores of lesions’ margin characteristics on TN, TP, FP, and FN cases.

Heatmap Score Range ≥ 0.5	BNet								GNet							
	Not Circumscribed				Circumscribed				Not Circumscribed				Circumscribed			
	TN	TP	FP	FN	TN	TP	FP	FN	TN	TP	FP	FN	TN	TP	FP	FN
Ablation-CAM	0.10	0.13	0.13	0.09	0.11	0.14	0.14	0.06	0.17	0.29	0.20	0.30	0.17	0.26	0.17	0.35
EGrad-CAM (top 95%)	0.07	0.07	0.08	0.06	0.09	0.10	0.08	0.06	0.15	0.17	0.14	0.19	0.16	0.20	0.11	0.24

**Table 7:** Results of evaluation with average drop (the lower the better), percent increase (higher is better), and win% (higher is better). The best result is bolded.

Models	Visualization Methods	Average % Drop in Confidence	Percent Increase in Confidence	Win %
BNet	Ablation-CAM	**35.04**	**39.80**	30.80
	EGrad-CAM (top 95%)	38.41	32.00	**38.60**
GNet	Ablation-CAM	31.38	**23.20**	**38.20**
	EGrad-CAM (top 95%)	**28.83**	22.00	35.60

## Data Availability

The datasets used in this study originated from two local hospitals in China. The datasets are not publicly available due to restrictions of the respective ethics agreements between the sponsor and the hospitals.
